# Supplementary material for: Central and peripheral clocks are coupled by a neuropeptide pathway in Drosophila
Source: Nat Commun. 2017 May 30;8:15563. doi: 10.1038/ncomms15563 (PMC5459987; doi:10.1038/ncomms15563)
Supplement: Supplementary Information — Supplementary Figures, Supplementary Table, Supplementary Methods and Supplementary References. [file ncomms15563-s1.pdf]

SUPPLEMENTARY METHODS

Fly stocks

All flies were reared at room temperature (21-25°C) on standard cornmeal/molasses/yeast food medium. All UAS and gal4 stocks have previously been described; unless noted, they were obtained from the Bloomington *Drosophila* stock center (Bloomington, Indiana, USA) or the Vienna *Drosophila* Resource Center (VDRC; Vienna, Austria). They included: wildtype (Canton-S strain), *white*[1118], *tim*-gal4 [1]*; ptth*-gal4 (45A3,117b3) [2]; *phm*-gal4, UAS-*GFP*/TM6B [3]; *pdf*-gal4; *da*-gal4; *elav*-gal4; cre-luc [4]; *per69x3-luc* [5]; UAS-*han* (UAS-PDFR; [6]; UAS-*cyc[Δ172]* and UAS-*cyc[Δ901]* [7]; UAS-*dbt*[S] and UAS-*dbl*[L] [8]; UAS-*dORK-ΔC1* and UAS-*dORK-ΔNC1* [9]; UAS-*grim* [10]; UAS-Kir2.1 [11, 12]; UAS-*eko* [13]; UAS-*IVS-myr-GFP* [14], UAS-*epac1-camps*(50A)[15]; 20xUAS-*IVS-GCaMP6m* [16]; UAS-*dicer2*, UAS-*torso* RNAi (VDRC36280 and VDRC109108), UAS-*raf* RNAi (VDRC107766), UAS-*erk* RNAi (VDRC109573), UAS-*ras* RNAi (VDRC106642), UAS-*Ptth* RNAi (VDRC #102043), UAS-*sNPFR*; UAS-*sNPFR* RNAi [17], UAS-*PDFR* RNAi (VDRC106381), *pdf*-LexA [18].

SUPPLEMENTARY FIGURES

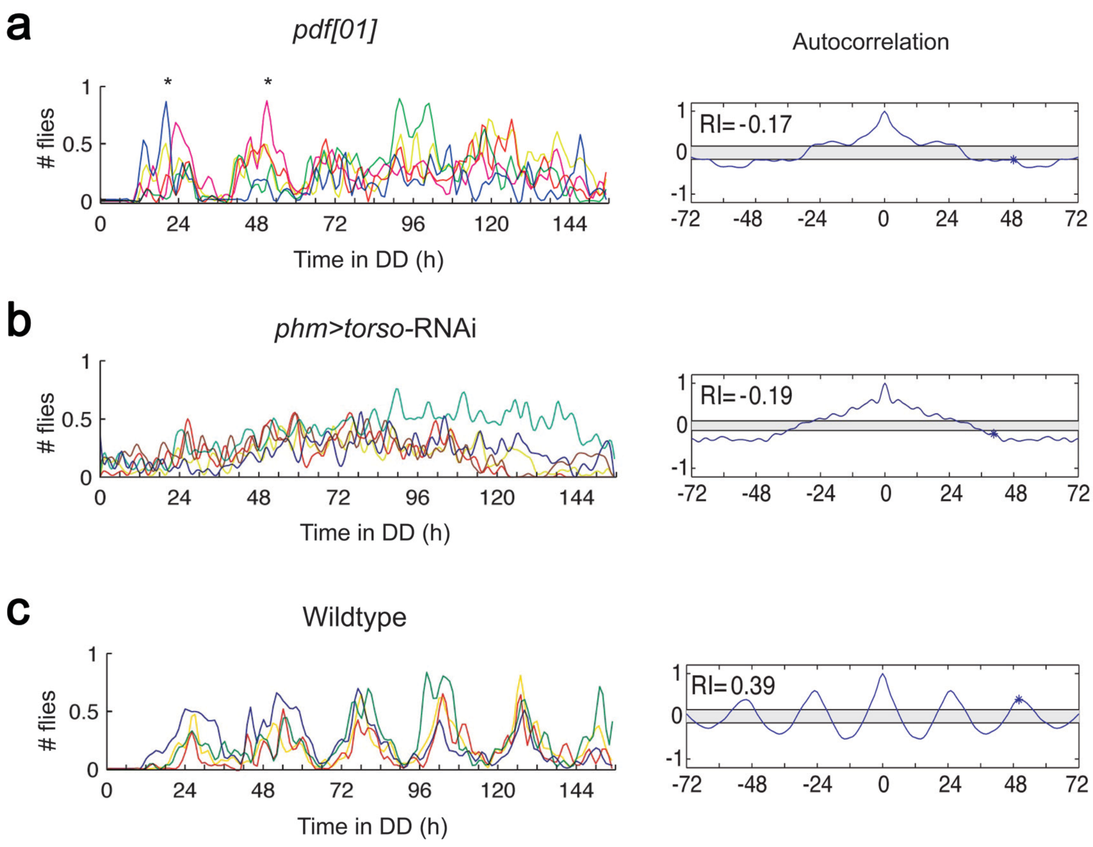

Supplementary Figure 1. Residual rhythmicity persists in *pdf* null mutants but not after *torso* knockdown in the PG. **(a-c)** Left: Superimposed traces of pattern of eclosion in DD obtained for cultures of *pdf*^{01} null mutants **(a)**, *torso* knockdown in the PG **(b)**, and wildtype controls **(c)**. Right: autocorrelation analysis of representative record, with RI value indicated. Cultures of *pdf* null allele shows residual circadian rhythmicity of emergence: although globally arrhythmic, clear peaks of emergence are apparent during the first 2 days of DD (a; asterisks); no such rhythmicity is seen when *torso* is knocked

down in the PG (b). Number of separate experiments displayed: 5 (for a and b) and 4 (for c). For (b) RNAi knockdown was enhanced by co-expression of *dcr2*.

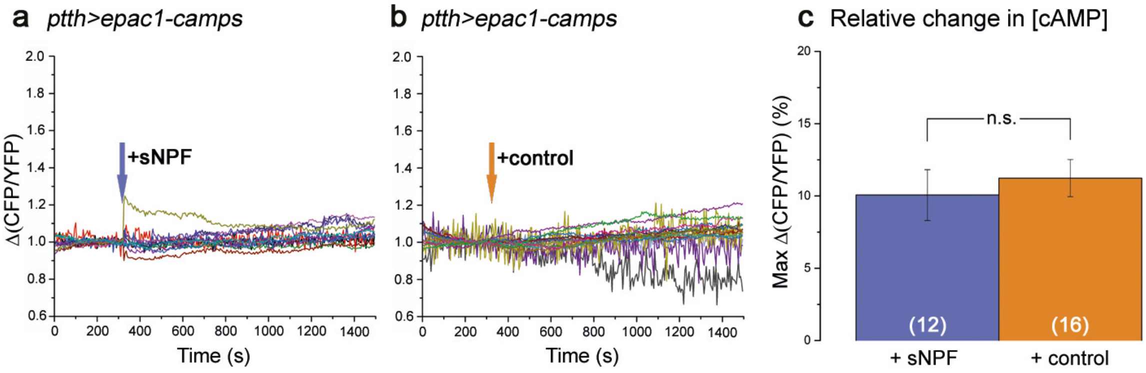

Supplementary Figure 2. PTTH neurons do not change cAMP levels in response to sNPF. **(a, b)** Bath application of sNPF (10^{-5} M) in HL3.1 containing 0.1% DMSO **(a)** or HL3.1 containing 0.1% DMSO **(b)** did not elicit changes in cAMP levels in PTTH neurons expressing Epac1-camps cAMP sensor. **(c)** Summary of results shown in (a) and (b), indicating average of the maximal response after sNPF application (± SEM). Each trace in (a) and (b) represents the response from a single ROI (N= 12 ROIs from 8 pharate adult brains for (a), 16 ROIs from 10 pharate adult brains for (b)). (n.s.= p>0.05; Wilcoxon rank sum test).

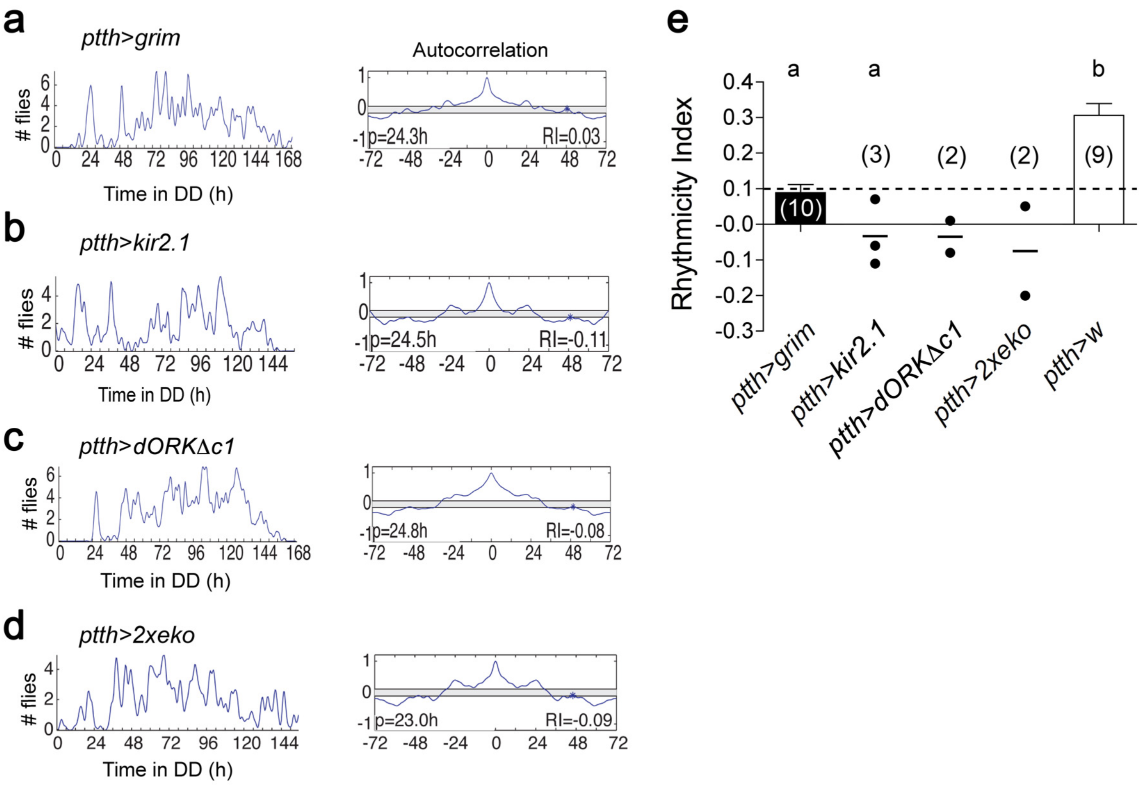

Supplementary Figure 3. Targeted killing or silencing of PTTH neurons renders arrhythmic the pattern of adult emergence. **(a-d)** Left: pattern of emergence of flies in which PTTH neurons were selectively killed **(a)** or electrically silenced (**b-d)**; Right: corresponding autocorrelation analysis with value of RI indicated. **(e)** Average RI (± SEM) for genotypes shown in (a-d); individual values are indicated when N<5 and average indicated by short horizontal line. Dashed line marks RI cutoff value of 0.1. Different letters indicate statistically significant differences ($p<0.05$; one-way ANOVA, Tukey's *post-hoc* multiple comparison analyses); this analysis did not include genotypes shown in (c) and (d) because N=2. Numbers in parenthesis indicate number of separate experiments.

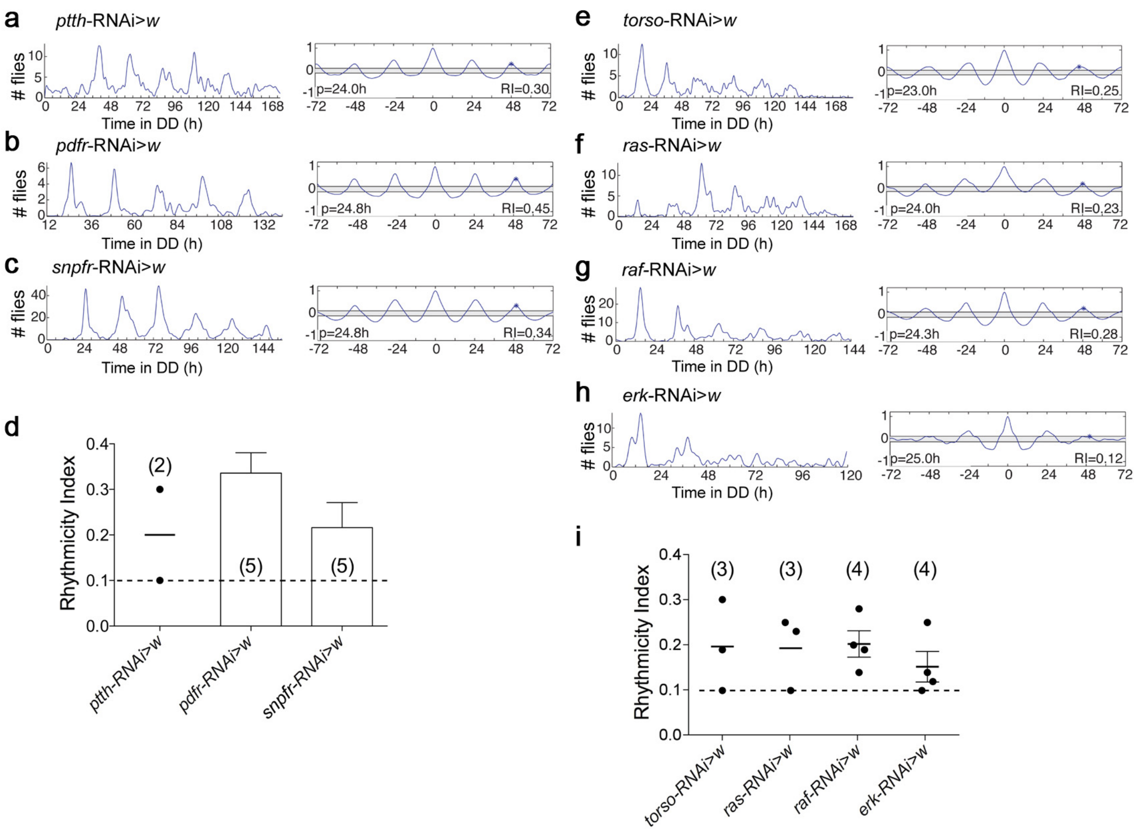

Supplementary Figure 4. Flies bearing single UAS-RNAi insertion express normal circadian rhythmicity of eclosion. **(a-c)** Eclosion rhythmicity phenotypes of flies heterozygous for UAS-RNAi lines: PTTH **(a)**, PDFR **(b)**, and sNPFR **(c)**. Left: pattern of eclosion in DD; right: corresponding autocorrelation analysis with dominant periodicity and value of RI indicated. **(d)** Average RI (± SEM) for genotypes shown in (a-c); individual values are indicated when N<5 and average indicated by short horizontal line. Dashed line marks RI cutoff value of 0.1. Numbers in parenthesis indicate number of separate experiments. **(e-h)** Eclosion rhythmicity phenotypes of flies heterozygous for UAS-RNAi lines: *torso* **(e)**, *ras* **(f)**, *raf* **(g)**, and *erk* **(h)**. Left: pattern of eclosion in DD; right: corresponding autocorrelation analysis with dominant periodicity and value of RI

indicated. **(i)** Average RI (± SEM) for genotypes shown in (e-h); individual values are indicated because N<5; average indicated by short horizontal line (SEM is indicated when N=4). Dashed line marks RI cutoff value of 0.1. Numbers in parenthesis indicate number of separate experiments.

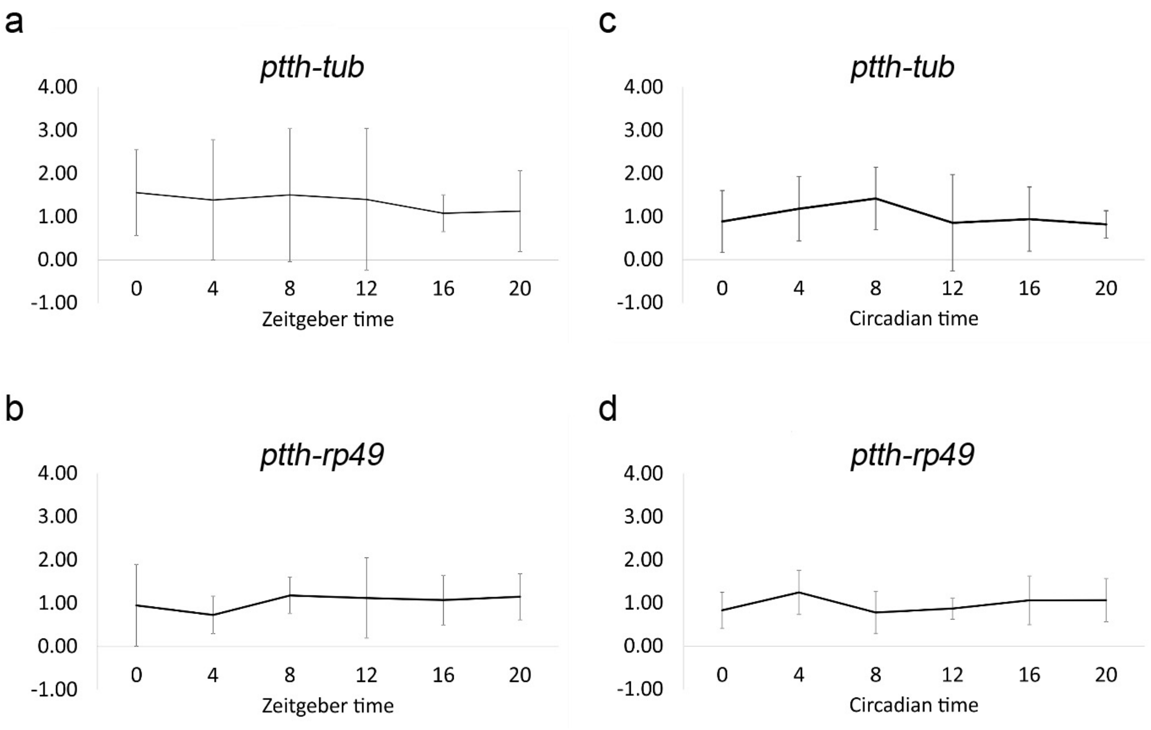

Supplementary Figure 5. Temporal expression of *Ptth* mRNA in heads of pharate adult flies after LD12:12 entrainment. Relative expression levels of *Ptth* mRNA under LD **(a, b)** and DD **(c, d)** conditions quantified by qPCR using *α-tubulin* (a, c) and *rp49* (b, d) as reference genes. Comparison of the expression levels between the various time points by one-way ANOVA revealed no significant differences ($p>0.05$). Bars represent standard deviation. N= 5, measured with three technical replicates each.

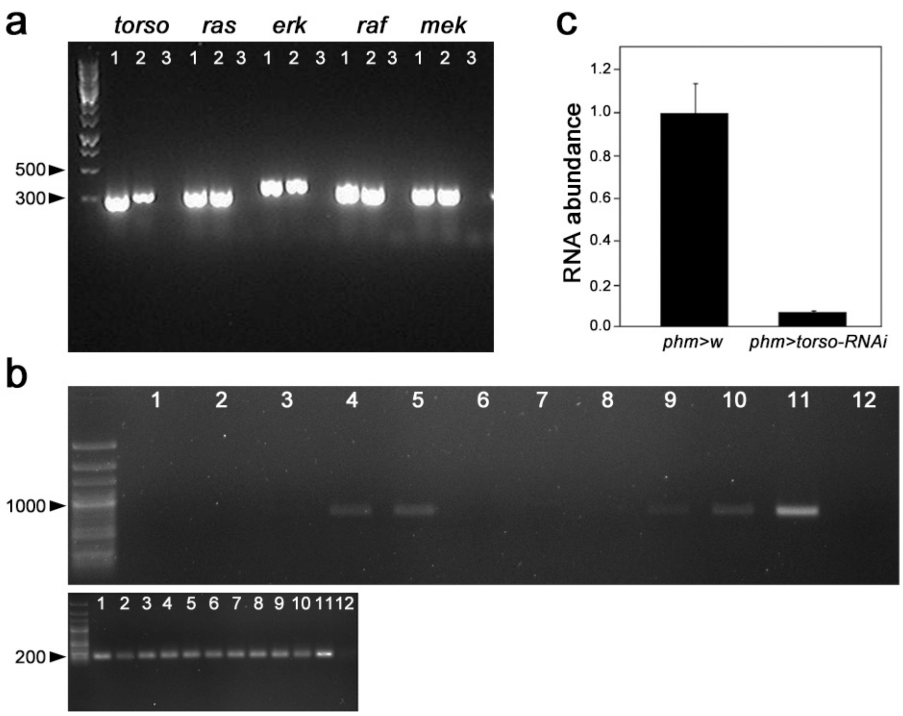

Supplementary Figure 6. Expression of *torso* and of genes downstream of *torso*. (**a**) *torso*, *ras*, *erk raf*, and *mek* (cf., Fig. 3a) can be amplified from whole prepupae (lanes labeled 1) and also from dissected PG (lanes labeled 2), showing that transcripts for each of these genes are present in the PG. Lanes labeled 3: no cDNA control. **(b)** Top gel: RTPCR for *torso* using RNA from pharate adults (lanes 1-5), adults (lanes 6-10), and wandering III instar larvae (lane 11). Lower gel: α-tubulin was amplified as control. Lanes 1 and 6: whole brain; lanes 2 and 7: optic lobes; lanes 3 and 8: retina; lanes 4 and 9: female gonads; lanes 5 and 10, male gonad; lane 11, larval ring gland; lane 12, negative control (no cDNA added). These analyses show that the *torso* receptor is also expressed in female and male gonads, as well as in the ring gland of III instar larvae, but not in the pharate CNS. **(c)** qRTPCR for *torso* using RNA from PG of control prepupae (left) and from prepupae expressing *torso* RNAi in PG (right); differences were

statistically significant ($p<0.05$; *t*-test). N=3 independent replicates, each with three technical replicates each. For (a) and (b) numbers to the left of gels indicate size of PCR products, in base pairs. For (c) RNAi knockdown was enhanced by co-expression of *dcr2*.

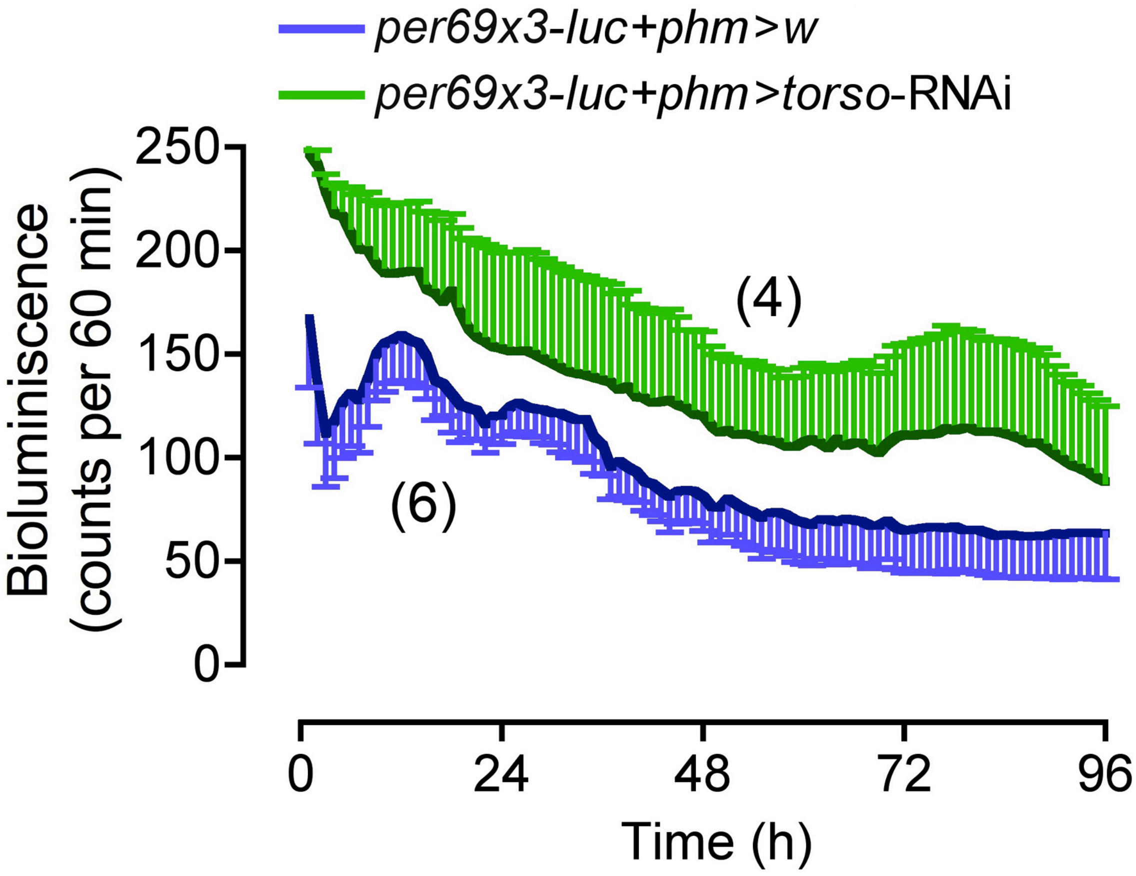

Supplementary Figure 7. Knockdown of *torso* expression in the PG eliminated circadian fluctuations of *period*-driven bioluminescence in the PG. Values are averages ± SEM; numbers in parenthesis indicate number of records averaged. All genotypes expressed *dcr2* to enhance effectiveness of RNAi knockdown.

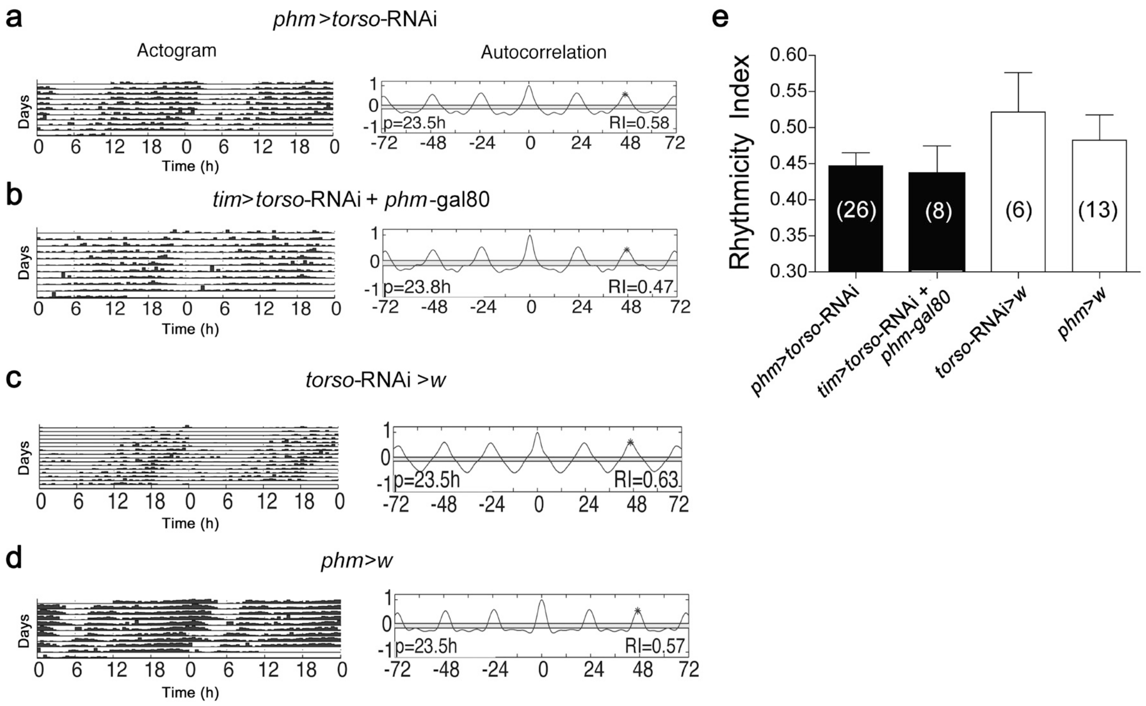

Supplementary Figure 8. Interfering with *torso* signaling does not affect rhythmicity of adult locomotor activity. **(a, b)** Expressing *torso* RNAi in the PG **(a)** or all clock cells except the PG **(b)** does not affect rhythmicity of adult locomotor activity. **(c, d)** Locomotor activity records of corresponding controls. Left: actogram record of locomotor activity of single fly in DD; right: corresponding autocorrelation analysis, with dominant periodicity and value of RI indicated. **(e)** Average RI (± SEM) for genotypes shown in (a and b) and for controls (c and d); no statistically significant differences were detected between the different genotypes ($p>0.05$; one-way ANOVA, Tukey's *post-hoc* multiple comparison analyses). Numbers in parenthesis indicate number of separate experiments. In all experiments RNAi knockdown was enhanced by co-expression of *dcr2*.

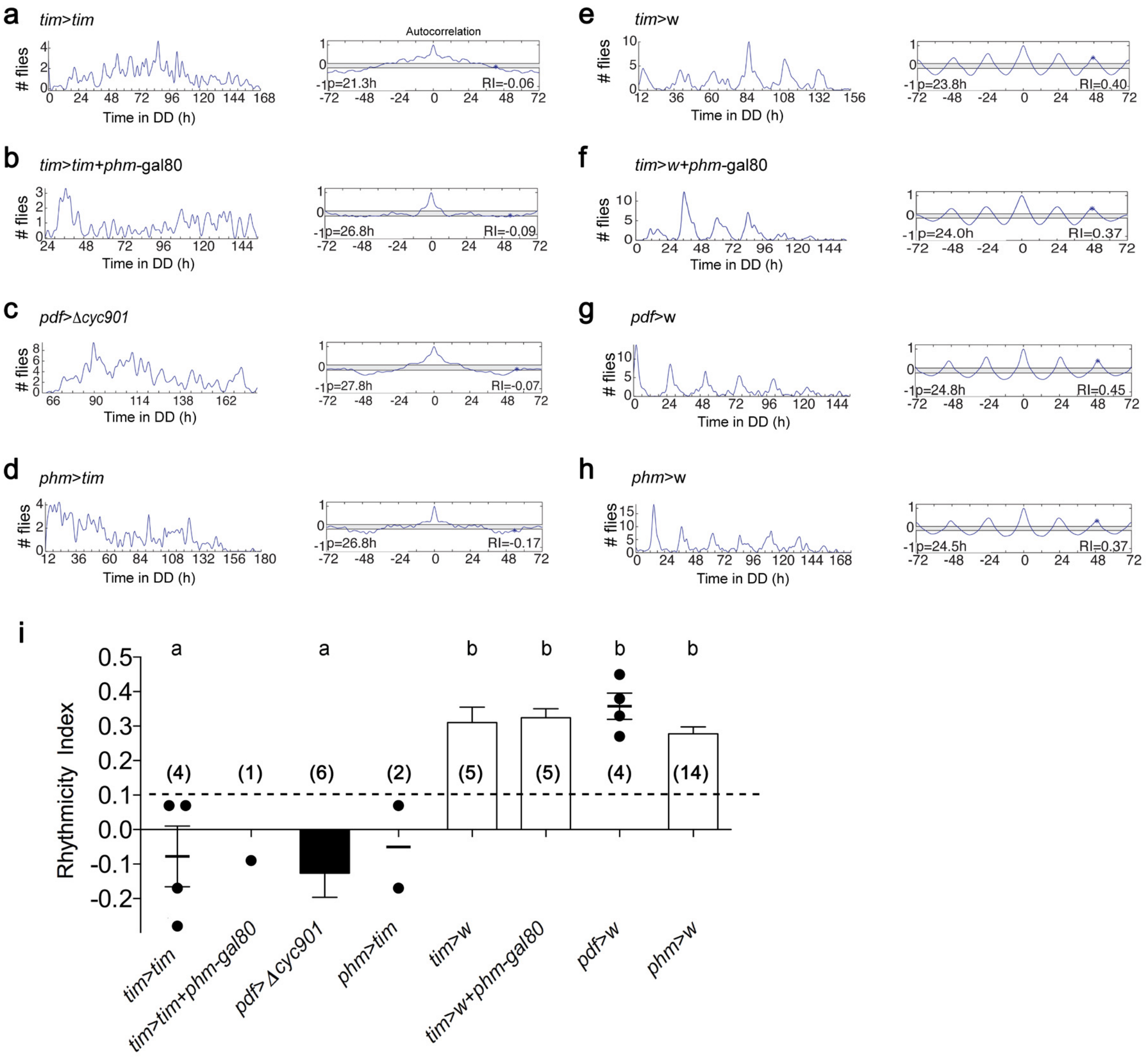

Supplementary Figure 9. Stopping all clocks, the brain clock, or the PG clock using other approaches renders arrhythmic the pattern of adult emergence. (a) Stopping all clocks by overexpressing *tim* using *tim*-gal4 driver eliminates the circadian rhythm of emergence. Left: pattern of eclosion in DD; right: corresponding autocorrelation analysis, with RI value indicated. **(b,c)** Stopping the brain clock by either overexpressing *tim* using *tim*-gal4 + *phm*-gal80 driver **(b)** or expressing the dominant negative *cycle* allele, *cyc[Δ901]*, in PDF neurons **(c)** renders arrhythmic the

pattern of adult emergence. (Strictly speaking, in (b) gene expression is driven in all clocks except the PG. However, since circadian rhythmicity of emergence depends only on the clocks in the brain and in the PG, such experiment is equivalent to driving gene expression only in the brain.) **(d)** Stopping the PG clock by overexpressing *tim* in the PG eliminates the circadian rhythmicity of emergence. **(e-h)** Controls bearing only the gal4 driver express a normal circadian rhythmicity of emergence. **(i)** Average values of RI ($\pm$ SEM) for genotypes shown in (A-D); individual values are indicated when N<5 and average indicated by short horizontal line (SEM also indicated when N=4). Dashed line marks RI cutoff value of 0.1. Numbers in parenthesis indicate number of separate experiments. Different letters indicate statistically different groups ($p<0.05$; one-way ANOVA, Tukey's *post-hoc* multiple comparison analyses); genotype shown in (b) and (d) were not included in this analysis because n<3.

Supplementary Table 1. PCR Primers.

Gene	Sequence of primer pair	Product size (bp)	Use
phm[1]	TAGATCTTGATGCGCTGCT		Subcloning of 5' *phm* region
	CACTTTCGATTTCCTCCTGC	1200	Subcloning of 5' *phm* region
ChR2-XXL[2]	GGGGACAAGTTTGTACAAAAAAGCAGG-CTGGGCCTAGGTACCTCGAGCTCTAG		Gateway-cloning
	GGGGACCACTTTGTACAAGAAAGCTGG-GTAACACAAAGATCCTCTAGTACCGG		Gateway-cloning
torso[1]	AAGCCCCGAATACCACTGAAAT		RTPCR
	AGCACACCAAAGGACCAAACAT	302	RTPCR
ras[1]	CGAGGACTCTTACCGAAAGCAA		RTPCR
	TTGGCGGATGTCTCAATGTATG	333	RTPCR
raf[1]	CCTCTTCATGGGCTGTGTATCC		RTPCR
	ACTCCGCCAACAGTTCGTACAT	416	RTPCR
mek[1]	AGATCTGTGATTTCGGCGTCTC		RTPCR
	TGTTTCTTCAGGCAGATGTCCA	373	RTPCR
erk[1]	GAAGGAGCTTATGGCATGGTTG		RTPCR
	GATCTGCAATACGAGCCAATCC	430	RTPCR
rp49[1]	CCCAAGGGTATCGACAACAGAG		RTPCR
	GACAATCTCCTTGCGCTTCTTG	224	RTPCR
torso[2]	TCATCGAGAGGGCAACATGG		RTPCR
	CACAGTGGACAGCATCGAGT	667	RTPCR

α-tubulin[2]	TCTGCGATTCGATGGTGCCCTTAAC		RTPCR
	GGATCGCACTTGACCATCTGGTTGGC	198	RTPCR
torso[1]	CCAGTGATCTCTTGCAGCTAC		QRTPCR
	AGTCTGTGTTTAAGGGCGG	145	QRTPCR
rp49[1]	TGTGATGGGAATTCGTGGG		QRTPCR
	ATCTTGGGCCTGTATGCTG	212	QRTPCR
α-tubulin[2]	TTTACGTTTGTCAAGCCTCATAG		QRTPCR
	AGATACATTCACGCATATTGAGTTT	149	QRTPCR
rp49[2]	ATGCATTAGTGGGACACCTTCTT		QRTPCR
	ATGCATTAGTGGGACACCTTCTT	130	QRTPCR
Ptth[2]	AAAGGTAATCCGAGAGGCGG		QRTPCR
	ATAATGGAAATGGGCAACCACG	136	QRTPCR

[1] Primers used by Ewer lab

[2] Primers used by Wegener lab.

SUPPLEMENTARY REFERENCES

1. Kaneko M, Hall JC. Neuroanatomy of cells expressing clock genes in *Drosophila*: transgenic manipulation of the *period* and *timeless* genes to mark the perikarya of circadian pacemaker neurons and their projections. *J Comp Neurol* **422**, 66-94. (2000).

2. McBrayer Z*, et al.* Prothoracicotropic hormone regulates developmental timing and body size in *Drosophila*. *Dev Cell* **13**, 857-871 (2007).

3. Mirth C, Truman JW, Riddiford LM. The Role of the Prothoracic Gland in Determining Critical Weight for Metamorphosis in *Drosophila melanogaster*. *Curr Biol* **15**, 1796-1807 (2005).

4. Belvin MP, Zhou H, Yin JC. The *Drosophila* dCREB2 gene affects the circadian clock. *Neuron* **22**, 777-787. (1999).

5. Stanewsky R. Analysis of rhythmic gene expression in adult *Drosophila* using the firefly luciferase reporter gene. *Methods Mol Biol* **362**, 131-142 (2007).

6. Hyun S*, et al. Drosophila* GPCR *Han* is a receptor for the circadian clock neuropeptide PDF. *Neuron* **48**, 267-278. (2005).

7. Tanoue S, Krishnan P, Krishnan B, Dryer SE, Hardin PE. Circadian clocks in antennal neurons are necessary and sufficient for olfaction rhythms in *Drosophila*. *Curr Biol* **14**, 638-649. (2004).

8. Yao Z, Shafer OT. The *Drosophila* circadian clock is a variably coupled network of multiple peptidergic units. *Science* **343**, 1516-1520. doi: 1510.1126/science.1251285. (2014).

9. Nitabach MN, Blau J, Holmes TC. Electrical silencing of *Drosophila* pacemaker neurons stops the free-running circadian clock. *Cell* **109**, 485-495. (2002).

10. Wing JP, Zhou L, Schwartz LM, Nambu JR. Distinct cell killing properties of the *Drosophila reaper*, *head involution defective*, and *grim* genes. *Cell Death Differ* **5**, 930-939 (1998).

11. Johns DC, Marx R, Mains RE, O'Rourke B, Marban E. Inducible genetic suppression of neuronal excitability. *J Neurosci* **19**, 1691-1697. (1999).

12. Baines RA, Uhler JP, Thompson A, Sweeney ST, Bate M. Altered electrical properties in *Drosophila* neurons developing without synaptic transmission. *J*

Neurosci **21**, 1523-1531. (2001).

13. White BH*, et al.* Targeted attenuation of electrical activity in *Drosophila* using a genetically modified K(+) channel. *Neuron* **31**, 699-711. (2001).

14. Pfeiffer BD*, et al.* Refinement of tools for targeted gene expression in *Drosophila*. *Genetics* **186**, 735-755. doi: 710.1534/genetics.1110.119917. Epub 112010 Aug 119919. (2010).

15. Shafer OT, Kim DJ, Dunbar-Yaffe R, Nikolaev VO, Lohse MJ, Taghert PH. Widespread receptivity to neuropeptide PDF throughout the neuronal circadian clock network of *Drosophila* revealed by real-time cyclic AMP imaging. *Neuron* **58**, 223-237 (2008).

16. Chen TW*, et al.* Ultrasensitive fluorescent proteins for imaging neuronal activity. *Nature* **499**, 295-300. doi: 210.1038/nature12354. (2013).

17. Lee KS, You KH, Choo JK, Han YM, Yu K. *Drosophila* short neuropeptide F regulates food intake and body size. *J Biol Chem* **279**, 50781-50789. Epub 52004 Sep 50721. (2004).

18. Shang Y, Griffith LC, Rosbash M. Light-arousal and circadian photoreception circuits intersect at the large PDF cells of the *Drosophila* brain. *Proc Natl Acad Sci U S A* **105**, 19587-19594 (2008).
